# Supplementary material for: Predicting visual function by interpreting a neuronal wiring diagram
Source: Nature. 2024 Oct 2;634(8032):113–23. doi: 10.1038/s41586-024-07953-5 (PMC11446822; doi:10.1038/s41586-024-07953-5)
Supplement: Supplementary file 6 — CRF and ERF predictions for individual TmY4 and TmY9 cells. Analogous to Supplementary Data 3, but for TmY target types. Shown are the top four monosynaptic pathways, the strongest pathway passing through each of the top ten intermediary types (ranking from Extended Data Fig. 7), and the trisynaptic pathway Tm1–TmY–Dm3–TmY (see the section entitled Prediction of spatial normalization). [file 41586_2024_7953_MOESM6_ESM.zip › DataS4/TmY9qΓèÑ/720575940632729764.pdf]

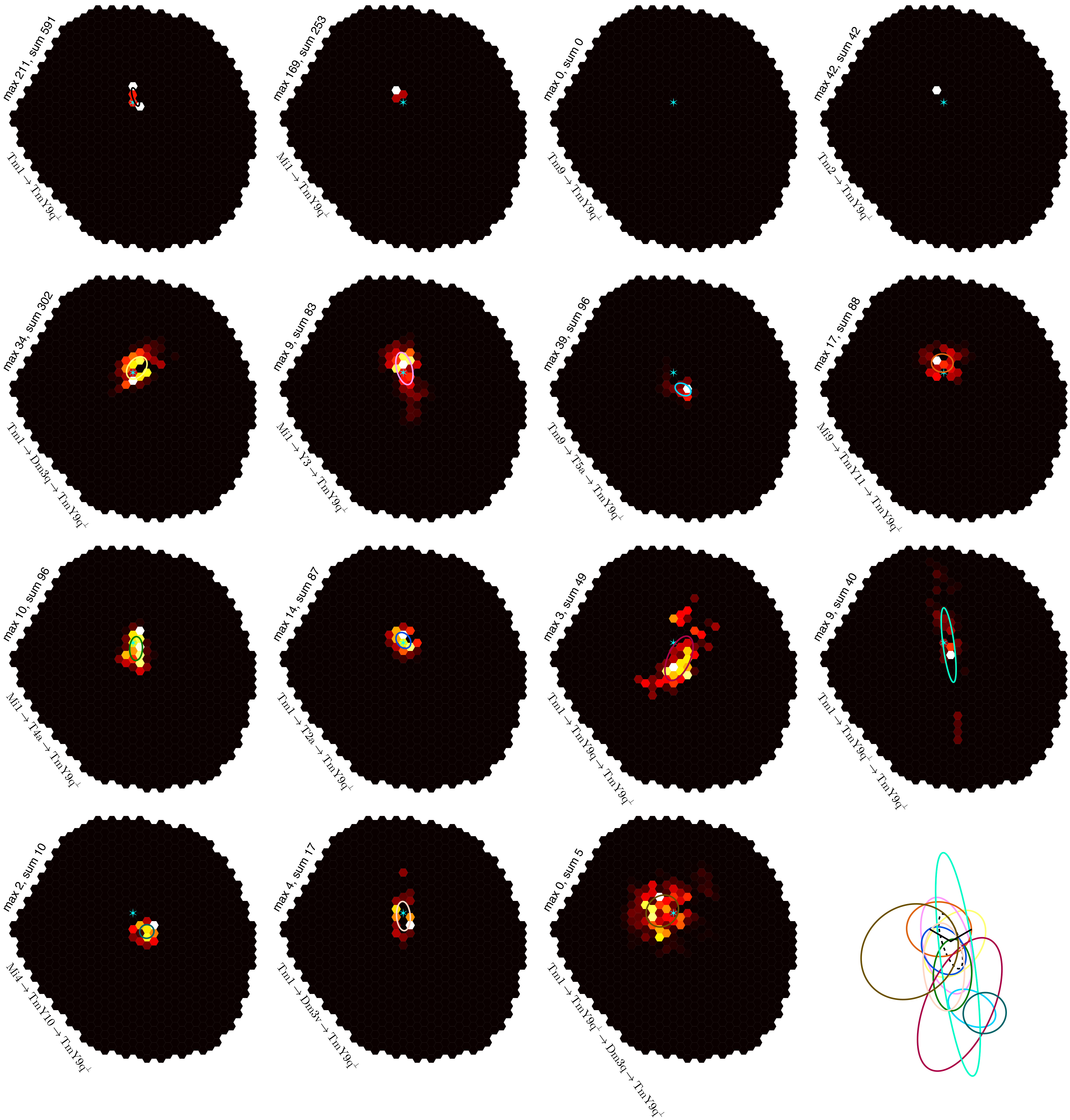
max 211, sum 591
Tm1 → TmY9q⊥
max 169, sum 253
Mi1 → TmY9q⊥
max 0, sum 0
Tm9 → TmY9q⊥
max 42, sum 42
Tm2 → TmY9q⊥
max 34, sum 302
Tm1 → Dm3q → TmY9q⊥
max 9, sum 83
Mi1 → Y3 → TmY9q⊥
max 39, sum 96
Tm9 → T5a → TmY9q⊥
max 17, sum 88
Mi9 → TmY11 → TmY9q⊥
max 10, sum 96
Mi1 → T4a → TmY9q⊥
max 14, sum 87
Tm1 → T2a → TmY9q⊥
max 3, sum 49
Tm1 → TmY9q⊥ → TmY9q⊥
max 9, sum 40
Tm1 → TmY9q⊥ → TmY9q⊥
max 2, sum 10
Mi4 → TmY10 → TmY9q⊥
max 4, sum 17
Tm1 → Dm3v → TmY9q⊥
max 0, sum 5
Tm1 → TmY9q⊥ → Dm3q → TmY9q⊥
